# The use of MRI in the diagnosis of ovarian cancer

**DOI:** 10.3389/fmed.2026.1889837

**Published:** 2026-08-25

**Authors:** Zhenglin Li, Kunlong Meng, Xinqiao Mao, Hengrun Ju, Yuying Dong, Tong Li, Wenjia Liu, Qianqian Li

**Affiliations:** 1 School of Radiology, Shandong First Medical University, Tai’an, Shandong, China; 2 Department of Nuclear Medicine, Shandong Provincial Hospital Affiliated to Shandong First Medical University, Jinan, Shandong, China; 3 Department of Oncology, The Affiliated Hospital of Shandong University of Traditional Chinese Medicine, Jinan, China; 4 Department of Obstetrics and Gynecology, Shandong Provincial Maternal and Child Health Care Hospital Affiliated to Qingdao University, Jinan, China

**Keywords:** deep learning, diagnosis, magnetic resonance imaging, ovarian cancer, precision medicine, radiomics

## Abstract

Ovarian cancer is a malignant tumor of the female reproductive system with a high mortality rate. Currently, China has the highest number of new ovarian cancer cases worldwide; therefore, preoperative diagnosis is of particular importance. Magnetic resonance imaging (MRI), with its excellent soft tissue resolution and absence of ionizing radiation risk, has become a key imaging modality for the non-invasive diagnosis of ovarian cancer in clinical practice. This article systematically reviews the latest research advances in the application of conventional morphological MRI, multiparametric MRI, and the integration of MRI with radiomics and deep learning technologies in the diagnosis and treatment of ovarian cancer. It focuses on their clinical value in assessing the benign or malignant nature of ovarian tumors, classifying pathological subtypes of epithelial and non-epithelial ovarian cancers, staging ovarian cancer, monitoring treatment efficacy, and predicting prognosis. Additionally, it outlines research directions for emerging technologies such as molecular probes. Existing studies have confirmed that multimodal MRI fusion technology can significantly improve the diagnostic accuracy of ovarian cancer; however, challenges remain, including a lack of model generalizability, insufficient interpretability, difficulty in detecting micro-lesions, and low clinical adoption rates. Future efforts should focus on establishing standardized MRI scanning protocols and data standards, conducting large-scale, multicenter prospective studies, deepening the integrated application of interpretable artificial intelligence and molecular imaging, and constructing an integrated “imaging-biology-clinical” precision assessment system to advance the development of precision preoperative diagnosis and treatment for ovarian cancer.

## 1 Introduction

**FIGURE 1:**
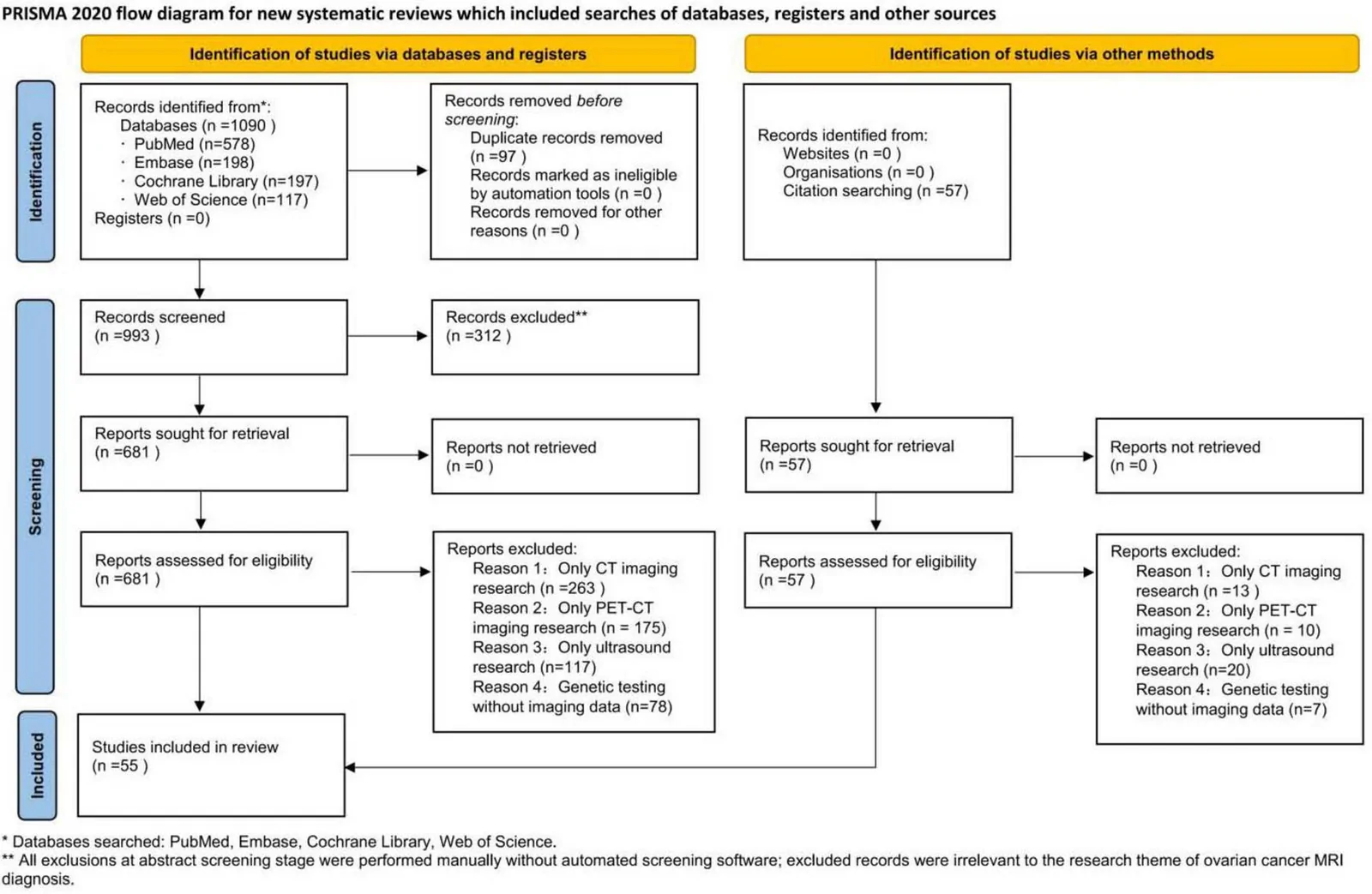
PRISMA 2020 flow diagram illustrating the literature screening process of this systematic review on MRI diagnosis of ovarian cancer. A total of 1090 records were initially retrieved from four electronic databases (PubMed, Embase, Cochrane Library, Web of Science), and an additional 57 studies were supplemented via citation tracking. After deduplication, title/abstract screening and full-text eligibility assessment, 55 qualified articles were finally included for qualitative and quantitative synthesis. All irrelevant literatures excluded at the abstract screening stage were manually filtered, with elimination criteria limited to studies relying solely on CT, PET-CT, ultrasound, or genetic testing without MRI imaging data.

In recent years, there has been a steady rise in the incidence of ovarian cancer, a female-specific cancer with a high mortality rate. As of 2021, China had the highest number of new ovarian cancer cases worldwide (1). Therefore, the importance of accurately identifying ovarian cancer prior to surgery continues to grow. MRI, which offers excellent soft-tissue contrast resolution and does not expose patients to radiation, is a crucial diagnostic tool for the non-invasive detection of ovarian cancer (2). The integration of technologies such as multi-sequence feature fusion networks, bioinformatics, and deep learning with MRI has made the diagnosis of ovarian cancer more accurate, and it is now possible to accurately identify different pathological subtypes, driving clinical workflows gradually toward precision medicine (3, 4). However, as emphasized by recent clinical feedback, significant challenges remain regarding the clinical translation of these emerging technologies, primarily manifested in low clinical adoption rates, limited model generalizability, and a lack of transparency in algorithmic decision-making. To address these issues, this review provides a balanced evaluation of current MRI techniques, distinguishing validated clinical applications from experimental concepts. This review aims to summarize the latest advances in MRI technology for the diagnosis of ovarian cancer, elucidate the relationships among different research methods, identify critical gaps in the field, and provide a concrete roadmap for future research (Figure 1 and Tables 1, 2).

**TABLE 1:** Comparison of typical MRI features of common ovarian tumors.

Tumor type	T2WI signal	DWI signal	ADC value	DCE features	Other typical findings
Benign tumors	Homogeneous low/intermediate signal	Isointense/low signal	High (>1.2 × 10^−3^ mm^2^/s)	No/mild enhancement	Well-defined border, thin and regular cyst wall
Malignant tumors	Heterogeneous high signal	Markedly high signal	Low (<1.0 × 10^−3^ mm^2^/s)	Marked heterogeneous enhancement	Solid components, thick cyst wall/nodules, ascites
HGSOC	Isointense/high signal (solid)	Markedly high signal	Markedly low (high cellular density)	Marked enhancement, often with necrosis	Large cystic-solid mass, peritoneal implants, massive ascites
CCC	Cystic-solid, often with mural nodules	Intermediate/high signal	Higher than HGSOC	Marked enhancement of mural nodules	Often associated with endometrioma
MOC (invasive)	Multicystic with viscous content	High signal (mucin restricts diffusion)	Intermediate/low	Wall/septal enhancement	Older age, ascites, wall nodules; “honeycomb sign” more common in borderline tumors
Granulosa cell tumor (GCT)	Solid, may appear “honeycomb-like”	High signal	Intermediate	Marked enhancement (high ERmax)	Estrogen-related symptoms, often with endometrial thickening
Mature cystic teratoma (MCT)	High signal of fat component (suppressed on fat-sat)	No restriction in fat component	Not applicable	No or mild enhancement	Fat, calcification, hair (Rokitansky nodule)
Immature teratoma (IT)	Heterogeneous with scattered fat	Restricted diffusion in solid components	Low	Enhancement of solid components	Large tumor, diffuse fat distribution, AFP may be normal

HGSOC, high-grade serous ovarian carcinoma; CCC, ovarian clear cell carcinoma; MOC, mucinous ovarian carcinoma; GCT, granulosa cell tumor; MCT, mature cystic teratoma; IT, immature teratoma. ADC reference values should be interpreted according to specific MRI scanner and *b*-value.

**TABLE 2:** Comparison of application value of different MRI techniques in diagnosis and treatment of cancer.

Technical category	Representative sequences/methods	Main application scenarios	Merit	Limitation
Morphological MRI	T1WI, T2WI, CE-T1WI	Differentiation of benign and malignant, classification, assessment of local invasion	Radiation-free, high soft tissue resolution, widely available	Relying on physicians, inability to quantify intratumoral heterogeneity
Functional/ multiparametric MRI	DWI, DCE, ADC Quantitative	Differentiate benign and malignant, predict chemotherapy response, and evaluate peritoneal dissemination	Provide microstructural and hemodynamic information	Lack of standardized scanning and analysis protocols
Imaging omics (Radiomics)	High-throughput feature extraction (T2WI, CE-T1WI, DWI)	Distinguish type I/II EOC, predict MT subtype, differentiate PMOC from MOMC	Quantifying intratumoral heterogeneity and finding naked invisible features	Poor model interpretability and lack of multicenter generalization
Deep Learning (DL)	CNN (e.g., ResNet-18), multi-sequence fusion network	Automated focus detection and classification to assist junior physicians	High diagnostic accuracy, end-to-end self-contained	Big data training is required, generalization ability is limited, and “black box” problems
Molecular imaging probe	A gadolinium-labeled vector targeting HER2; pH/glutathione responsive nanoprobe	Visualizing tumor targets *in vivo* to guide targeted/immunotherapy	Realize the integration of “imaging-therapy-immunity”	Still in preclinical/animal testing stage

CE-T1WI, contrast-enhanced T1-weighted imaging; DWI, diffusion-weighted imaging; DCE, dynamic contrast enhancement; ADC, apparent diffusion coefficient; EOC, epithelial ovarian cancer; MT, mesenchymal transition; PMOCs, primary mucinous carcinoma; MOMC, metastatic mucinous carcinoma; CNN, convolutional neural network.

## 2 Literature search methodology

### 2.1 Search strategy and database sources

A comprehensive electronic search was executed across four major international biomedical databases to capture relevant studies published up to the current year:

- PubMed (*n* = 578)
- Embase (*n* = 198)
- Cochrane Library (*n* = 197)
- Web of Science (*n* = 117)

This primary database search retrieved a total of 1,090 documents. To minimize the risk of missing relevant literature, an additional 57 documents were identified through external sources via citation searching.

### 2.2 Inclusion and exclusion criteria

The study selection followed a stringent, multi-stage filtering process based on the PRISMA 2020 framework to isolate high-quality, clinically relevant evidence focusing on MRI applications in ovarian malignancy:

➀ Deduplication: A total of 97 duplicate records were removed before screening, leaving 993 unique records for initial screening.

➁ Topic Relevance Exclusion: Title and abstract screening were performed manually to exclude records irrelevant to the research theme of ovarian cancer MRI diagnosis. This led to the removal of 312 documents, leaving 681 database records for detailed eligibility assessment.

➂ Eligibility and Modality Filtering: A total of 681 reports from databases and 57 reports from citation searching were sought and successfully assessed for eligibility (with 0 reports missing during retrieval). To maintain a strict focus on magnetic resonance workflows, documents relying on alternative diagnostic modalities or lacking imaging data were excluded (*n* = 633 from databases; *n* = 50 from citation searching) according to four pre-specified criteria:

Reason 1 (Only CT imaging research): Excluded 263 database records and 13 citation-searched records.

Reason 2 (Only PET-CT imaging research): Excluded 175 database records and 10 citation-searched records.

Reason 3 (Only ultrasound research): Excluded 117 database records and 20 citation-searched records.

Reason 4 (Genetic testing without imaging data): Excluded 78 database records and 7 citation-searched records.

### 2.3 Final included studies

Following this methodical exclusion protocol, 55 high-quality articles – comprising 48 articles from database searches and 7 articles from citation searching – were ultimately selected for qualitative and quantitative synthesis within this comprehensive review (Figure 1).

## 3 Distinguishing between benign and malignant tumors

There are significant morphological differences between benign and malignant ovarian tumors, which form the basis for morphological diagnosis by MRI. A systematic review by Wang et al. established the central role of morphological features in preoperative assessment (5). Building on this foundation, researchers have transformed subjective qualitative assessments into standardized quantitative analyses, and a mature quantitative assessment system has been established for the most common types of epithelial ovarian tumors. Babaoglu et al. further developed a standardized scoring system based on 13 morphological features, providing a practical tool for the standardized clinical application of morphological features (6). However, relying solely on morphological features for assessment results in significant heterogeneity across studies, making it difficult to fully meet the needs of precise clinical diagnosis. A multi-parameter MRI-based approach can address the limitations of conventional morphological feature-based diagnosis and significantly improve diagnostic performance.

### 3.1 Implementation of the O-RADS MRI risk stratification system

To address the significant heterogeneity and subjectivity observed across early studies, the American College of Radiology (ACR) introduced the Ovarian-Adnexal Reporting and Data System (O-RADS) MRI scoring system. This standardized system provides a highly reproducible framework for risk stratifying indeterminate adnexal masses, typically those categorized as uncertain on initial pelvic ultrasound evaluations. By classifying lesions from O-RADS MRI 1 (normal) to O-RADS MRI 5 (high risk of malignancy), it offers objective cutoff values based on the presence of solid tissue, wall irregularities, and functional perfusion dynamics (7). Recent prospective validations demonstrate that incorporating O-RADS MRI into the clinical routine reduces false-positive findings and significantly improves inter-observer agreement, providing an essential link between clinical scanning and structured management (8).

### 3.2 Integration of multiparametric MRI with ultrasound and biomarkers

A multi-parameter MRI-based approach effectively addresses the limitations of conventional morphological feature-based diagnosis and significantly improves diagnostic performance. A systematic review by van Nimwegen et al. confirmed that the diagnostic accuracy for distinguishing benign from malignant ovarian tumors is significantly improved when conventional MRI sequences are combined with diffusion-weighted imaging (DWI). The study also established reference ranges and clinical diagnostic cutoff values for the ADC values of benign and malignant lesions, noting that perfusion parameters from DCE-MRI can further enhance diagnostic accuracy beyond existing diagnostic protocols (9). Eftekhari Moghadam et al. further demonstrated that the diagnostic performance of DWI combined with T1-weighted contrast-enhanced imaging is significantly superior to that of ADC value measurement alone (10). Crucially, modern diagnostic paradigms emphasize that MRI should not be used in isolation but rather integrated within a multimodal diagnostic framework. In clinical workflows, indeterminate adnexal masses identified via pelvic ultrasound are referred for secondary evaluation with multiparametric MRI (7). Combining MRI morphological and functional features with serum biomarkers – such as Cancer Antigen 125 (CA-125) and Human Epididymis Protein 4 (HE4) – drastically enhances diagnostic accuracy (11, 12). This comprehensive multi-omics integration allows for the accurate differentiation of complex benign conditions (e.g., atypical endometriomas or fibromas) from borderline ovarian tumors (BOTs) and early-stage invasive malignancies, establishing a definitive strategy for high-risk patient screening.

### 3.3 Deep learning and machine learning solutions

Deep learning technologies, exemplified by convolutional neural networks (CNNs), offer new solutions to the problems of low diagnostic accuracy and heavy reliance on physician experience in traditional MRI. Numerous studies have demonstrated that such models significantly outperform radiologists in diagnostic accuracy and can improve the diagnostic skills of junior radiologists. In particular, the pioneering research by Robin Wang et al. was the first to demonstrate the core value of this class of models in large-scale datasets; the integrated model they developed, which combines imaging features with clinical variables, demonstrated diagnostic performance significantly superior to that of radiologists of varying experience levels (13). Subsequent scholars have developed specialized models targeting other clinical pain points, further expanding the clinical applicability of this technology:

The two-stage end-to-end model developed by Wang et al. enables fully automated diagnosis of ovarian lesions – from localization to characterization – significantly lowering the barrier to clinical adoption (14).

Jiang et al. validated the diagnostic performance of deep learning models based on non-contrast-enhanced MRI sequences, providing a non-invasive solution for patients who cannot undergo contrast-enhanced imaging (e.g., severe renal insufficiency) and helping to promote the adoption of this method in primary care settings (15).

In contrast, the multicenter studies by Li et al. and Wang et al. addressed the limitation of poor generalization observed in single-center studies. By utilizing large, multicenter datasets, these studies validated the model’s ability to distinguish borderline and malignant ovarian tumors, as well as its diagnostic performance and stability for early-stage ovarian cancer (16, 17).

### 3.4 Critical appraisal of current AI studies

Despite these impressive performance statistics, a critical evaluation reveals that the current literature is heavily biased toward reporting positive findings derived from idealized, retrospectively collected datasets. Most validated models fail when exposed to real-world clinical data due to variations in vendor hardware (e.g., 1.5T vs. 3.0T scanners), inconsistent field-of-view settings, and diverse acquisition protocols. Furthermore, the exclusion of complex borderline tumors from many training datasets falsely elevates model performance. Future research must mitigate these biases through the strict inclusion of consecutive, unselected clinical cohorts and rigorous out-of-distribution external validations.

## 4 Diagnostic classification of epithelial ovarian cancer (EOC)

### 4.1 Differentiation between type I and type II epithelial ovarian cancer

Based on a dichotomous model, epithelial ovarian cancer is classified into indolent Type I and aggressive Type II, which exhibit significant differences in molecular characteristics, clinical behavior, treatment response, and prognosis. Therefore, accurate preoperative differentiation is crucial for personalized treatment. Early studies primarily focused on differences in traditional MRI morphological features and functional parameters, but did not address intratumoral heterogeneity. To this end, Fan et al. incorporated the difference in apparent diffusion coefficients (dADC) to comprehensively quantify tumor heterogeneity and found that Type II dADC was significantly higher than Type I (18). To address the influence of subjective experience, Qian et al. were the first to develop a multi-parametric MRI-based radiomics model, demonstrating that the combined model performed better (19). To address issues related to sample size and generalizability, Jian et al. conducted a multicenter study. The “conjoint model” they developed – comprising a combination of four sequence features – demonstrated superior discriminatory performance compared to any single-sequence model, and they introduced an “occlusion test” to identify the key regions influencing the model’s decisions (20). Wei et al. developed a “Deep Learning Radiomics Nomogram (DLRN)” and demonstrated that the diagnostic performance of this multimodal, deep learning-based radiomics nomogram model was significantly superior to that of single-modality models. These findings indicate that by integrating information from different dimensions, multimodal fusion models can characterize the complex phenotypes of Type I and Type II tumors more comprehensively and accurately, representing a cutting-edge direction in this field (4).

From traditional MRI, which relies on visual inspection, to radiomics for quantifying tumor heterogeneity, and on to deep learning for the automatic extraction of deep features, research on MRI in the field of distinguishing Type I from Type II epithelial ovarian cancer has followed a clear path of technological evolution. Early studies laid the foundation for the application of MRI in assessing ovarian function parameters and established the research direction in this field; subsequent radiomics studies further refined multi-sequence fusion methods; and the latest deep learning and radiomics curve models have enhanced diagnostic performance through the integration of multimodal information, thereby charting the future course of development in this field.

### 4.2 High-grade serous ovarian cancer (HGSOC)

High-grade serous carcinoma (HGSOC) is the most common and most aggressive histological subtype of epithelial ovarian cancer, accounting for more than 70% of all ovarian cancer deaths. Clinically, most cases are diagnosed at an advanced stage (FIGO stage III–IV).

Numerous previous studies have systematically described the typical MRI features of HGSOC. Cai et al. noted that, in pathological diagnosis, HGSOC often presents as a large cystic-solid mass with a high proportion of solid components, and most patients exhibit peritoneal seeding and massive ascites (21). Building on these findings, Saida et al. further discovered that HGSOC typically exhibits isointense or hyperintense signals on T2-weighted imaging (T2WI) sequences and markedly hyperintense signals on diffusion-weighted imaging (DWI) sequences. Additionally, the apparent diffusion coefficient (ADC) values are significantly lower than those of other subtypes of epithelial ovarian cancer, suggesting higher cellular density and reduced extracellular space, which is consistent with its pathological characteristics (22). The study by Jiang et al. demonstrated the added value of MRI from another perspective: it corrected the misclassification of high-grade serous ovarian cancer (HGSOC) by the ultrasound ADNEX model, achieving an accuracy rate of 80%. The study identified characteristic features for the auxiliary diagnosis of HGSOC on MRI, including a predominantly lobulated, solid, or cystic-solid mass with marked enhancement, often accompanied by necrosis. HGSOC exhibits significant heterogeneity at the histological and molecular levels, with the mesenchymal transition (MT) subtype being closely associated with poorer surgical outcomes, higher rates of residual disease after surgery, and sensitivity to specific chemotherapy regimens. To address the clinical need for precise identification of the MT subtype, imaging research has evolved from the analysis of functional parameters to the identification of high-throughput radiomic features (23). Cai et al. were the first to apply DWI histogram analysis to investigate its value in predicting MT HGSOC. The study found that ADC histogram parameters in patients with the MT subtype were significantly lower than those in patients with non-MT subtypes. A combined predictive model based on these parameters achieved an AUC of 0.82, providing the first evidence that the distribution characteristics of ADC values can indirectly reflect differences in tumor cell density and microstructure (21). To more comprehensively quantify tumor heterogeneity, Lin et al. conducted a radiomics study based on multi-sequence MRI (T2WI and CE-T1WI) and identified seven textural features; a random forest-based model demonstrated the best diagnostic performance (24).

Currently, MRI research on HGSOC has progressed from macroscopic morphological descriptions to the identification of molecular subtypes and the analysis of microenvironmental characteristics. However, it remains challenging to determine molecular subtypes directly based on imaging features. As medical research continues to advance, the application of multimodal fusion and deep learning technologies has further improved the diagnostic performance of these models.

### 4.3 Clear cell carcinoma of the ovary (CCC)

Clear cell carcinoma (CCC) is a subtype of epithelial ovarian cancer characterized by unique clinical and pathological features. Given its low sensitivity to platinum-based chemotherapy and frequent association with endometriosis, the accurate preoperative identification of CCC using imaging modalities is of great significance. In recent years, MRI research on CCC has evolved from macroscopic morphological descriptions to multi-parameter quantitative analysis and, subsequently, to the development of multimodal models.

Early studies focused on identifying the characteristic MRI morphological features that distinguish CCC from other subtypes of ovarian cancer. Mori et al. found that the ADC values for CCC were significantly higher than those for endometrioid carcinoma and serous carcinoma (25). Specifically for ovarian cancer, Takeyama et al. developed a model that combines MRI and radiomic features, which demonstrated the best diagnostic performance (26). Du et al. developed a deep learning network based on the fusion of multi-parametric MRI features from multiple sequences to distinguish between high-grade serous ovarian cancer (HGSOC) and clear cell carcinoma (CCC) preoperatively; the test results outperformed those of any single-sequence model. The latest research trend involves combining MRI assessment with standardized reporting systems to enhance the clinical applicability of the model (3). Lin et al. focused on high-risk masses with O-RADS scores of 4–5 and developed a predictive model that integrates clinical and MRI features, marking an important step toward clinical translation (27).

In summary, research on MRI in the differential diagnosis of clear cell ovarian carcinoma has followed a clear evolutionary trajectory: from qualitative to quantitative analysis, from single-parameter to multimodal fusion, and from technical exploration to clinical translation. Future research should continue along the current path of clinical translation, further integrating deep learning features to analyze image information from multiple dimensions and improve diagnostic performance.

### 4.4 Mucinous ovarian carcinoma (MOC)

Ovarian mucinous tumors encompass a spectrum of diseases ranging from benign lesions to invasive carcinomas; distinguishing between borderline tumors (MBOT) and invasive carcinomas (MOC) is of the greatest clinical significance.

Early studies focused on morphological features in conventional MRI. Hasbay et al. found that patients in the MOC group were older and more likely to present with ascites and wall nodules, whereas the “honeycomb sign” was more common in the MBOT group; however, there was significant overlap in the MRI findings between the two groups (28). To overcome this limitation, Araki et al. combined serological markers with tumor volume measurements and found that serum CA72-4 levels in MOC patients were significantly higher than those in the MBOT group. When CA72-4 was used in conjunction with tumor volume measured via three-dimensional MRI, the diagnostic performance (with an AUC of 0.875) was superior to that of either marker alone, providing the first evidence of the value of multidimensional information fusion (29). To overcome this limitation, Araki et al. combined serological markers with tumor volume measurements and found that serum CA72-4 levels in MOC patients were significantly higher than those in the MBOT group. When CA72-4 was used in conjunction with tumor volume measured via three-dimensional MRI, the diagnostic performance (with an AUC of 0.875) was superior to that of either marker alone, providing the first evidence of the value of multidimensional information fusion.

Regarding the differentiation between primary (PMOC) and metastatic (MOMC) cases, the decision tree developed by Cai et al. demonstrated good diagnostic performance (30). The results of the radiomics profiles developed by Shi et al. based on multi-parameter MRI were the best (31).

The MRI diagnosis of mucinous tumors has undergone a clear technological evolution: the differentiation between MBOT and MOC has progressed from a comparison of macroscopic features to the use of multidimensional models; meanwhile, the differentiation between PMOC and MOMC has evolved from morphological assessment and semi-quantitative analysis of cystic homogeneity to multi-parameter bioinformatics analysis, significantly improving diagnostic accuracy by mining deep image features.

## 5 Differential diagnosis of ovarian non-epithelial tumors

Ovarian non-epithelial tumors are classified into two main categories: sex cord-stromal tumors (OSCTs) and germ cell tumors (GCTs). However, these tumors exhibit a wide variety of subtypes, with significant differences in pathological characteristics and clinical manifestations; therefore, accurate preoperative differentiation is crucial for developing personalized treatment plans. In recent years, with continuous advancements in MRI technology – combined with the application of multimodal fusion, radiomics, and deep learning – the ability to distinguish between different tumor subtypes has significantly improved.

### 5.1 Differentiation of leydig-stromal tumor subtypes

The MRI findings of various subtypes of OSCTs overlap, and early studies have largely relied on clinical and morphological features rather than imaging features. Zhang et al. developed a clinical-imaging integrated model and found that clinical symptoms, signal intensity of solid components on T2-weighted imaging (T2WI), the honeycomb sign, and ADC values are independent factors for distinguishing granulosa cell tumors (GCTs) from other sex cord-stromal tumors (32). Regarding subtypes dominated by fibrous components, Cheng et al. compared and found that both the ADC values and maximum enhancement rate (ERmax) of GCTs were significantly higher than those of fibromas and fibrous theca lomas (33). Wu et al. introduced histogram analysis and found that variance parameters in DWI sequences were significantly superior to traditional ADCmin in distinguishing GCT from other OSCTs, demonstrating that texture analysis can more comprehensively capture tumor microheterogeneity and overcome the subjectivity of traditional region-of-interest measurements (34). Bo et al. were the first to systematically differentiate themofibroids with cystic changes from ovarian adenofibroids, providing a solution to the clinical challenge of distinguishing these two conditions in the absence of imaging modalities (35).

### 5.2 Differentiation of germ cell tumor subtypes: mature and immature teratomas

The study by Akcay et al. highlights the critical role of MRI in differentiating immature teratomas (IT). The study found that ITs exhibit the following typical MRI features: large tumor volume, scattered fatty components, and enhanced solid components, with the solid components often accompanied by restricted diffusion. Compared with mature cystic teratomas (MCT), the fatty distribution in ITs is more diffuse. It is worth noting that the currently common diagnostic approach relying solely on serum markers may have limitations. In this study, alpha-fetoprotein (AFP) levels were normal in all cases, indicating that the disease cannot be diagnosed based on biochemical markers alone; therefore, a comprehensive MRI evaluation is essential (36).

### 5.3 Differentiation between germ cell tumors and sex cord-stromal tumors

A review by Mitchell et al. noted that the presence of fat is a key indicator for distinguishing germ cell tumors (particularly mature cystic teratomas) from OSCTs, and fat suppression techniques and chemical shift imaging are important methods for visualizing macroscopic or microscopic fat within the tumor. Furthermore, unlike malignant germ cell tumors such as dysgerminomas, which present as lobulated solid masses with low-signal fibrous and vascular septa, OSCTs exhibit homogeneous low signal intensity, providing a systematic MRI differentiation framework for clinical practice (37). Building on this, Zheng et al. developed a CNN model based on ResNet-18 combined with deep learning technology, achieving high-precision differentiation between theca lutein cysts and solid ovarian cancer. Through Grad-CAM visualization, the model’s areas of focus were found to align with key pathological features such as the capsule and necrosis, thereby enhancing the model’s interpretability and clinical reliability (38).

In summary, the application of MRI in the differential diagnosis of non-epithelial ovarian tumors is no longer limited to traditional morphological descriptions; it has gradually evolved toward the combined use of multimodal quantitative analysis, radiomics, and deep learning technologies. In the future, multicenter validation and multi-omics integration will be key directions for improving diagnostic accuracy.

## 6 The use of MRI in the staging of ovarian cancer and predicting surgical resectability

### 6.1 Assessment of local invasion and peritoneal dissemination

Accurately assessing the extent of peritoneal dissemination in ovarian cancer to predict the feasibility of surgery can help avoid unnecessary open surgeries and thereby reduce postoperative complications. However, CT remains limited in assessing peritoneal dissemination due to practical clinical issues such as insufficient soft tissue resolution and extremely poor detection of microscopic peritoneal implants. A prospective head-to-head study by Kandemir et al. confirmed that CT has a sensitivity of only 38% and an AUC of only 0.678 for detecting peritoneal millet-seed-sized implants smaller than 0.5 cm. To address these core limitations of CT, researchers have explored the application of multiparametric MRI in this context. Currently, the value of MRI in assessing peritoneal dissemination is supported by high-level evidence-based data (39). The multicenter, prospective MISSION trial conducted by Berardi et al. demonstrated good inter-observer agreement between mriPCI and intraoperative gold-standard PCI (ICC = 0.81), with an AUC of 0.76 for predicting complete tumor debulking. Following the intraoperative disclosure of MRI results, surgeons’ detection rates of lesions improved significantly, and surgical strategies were adjusted in 19% of patients as a result (40). A prospective cohort study by Lee et al. demonstrated that fPCI derived from DWI was highly correlated with intraoperative PCI (*r* = 0.757, *p* < 0.001); when combined with ADC values and FIGO staging, fPCI achieved a 92.5% accuracy rate in predicting incomplete debulking (AUC = 0.947) (41). A head-to-head study by Kandemir et al. confirmed that, for the detection of peritoneal implants ≥0.5 cm, MRI achieved a diagnostic sensitivity of 95.45% and an AUC of 0.974, significantly outperforming CT (84.85%) and PET/CT (86.36%); (39). A systematic review by Chiu et al. also showed that MRI has an overall diagnostic sensitivity of 92% for peritoneal metastases, which is significantly higher than the 68% for contrast-enhanced CT and the 80% for PET/CT (42). Regarding the assessment of overall tumor burden, a prospective study by Jónsdóttir et al. demonstrated that PET/MRI is significantly more accurate than DWI-MRI alone in evaluating the extent of peritoneal cancer. The median PCI score was found to be closer to the intraoperative surgical gold standard, making PET/MRI a valuable complement to conventional MRI (43). Currently, the primary limitation of MRI is its significantly inadequate diagnostic performance for peritoneal micrometastases smaller than 0.5 cm. Data from Kandemir et al. indicate that MRI has a sensitivity of only 40.49% for such lesions, showing no significant difference compared to CT or PET/CT (39). Furthermore, bowel motion artifacts and respiratory motion compromise detection rates in hidden anatomical areas, such as the root of the small bowel mesentery and the dome of the diaphragm.

### 6.2 Integrating AI and radiomics for predicting surgical resectability

To maximize patient survival, the therapeutic benchmark for advanced epithelial ovarian cancer is achieving complete cytoreductive surgery (R0 resection, with no visible residual disease). Traditional methods relying on qualitative clinical assessment or single PCI scoring are heavily dependent on institutional experience. Emerging studies indicate that integration of MRI with artificial intelligence and high-throughput radiomics represents an important and rapidly evolving field that deserves greater clinical attention (44).

Recent machine learning models utilize multi-sequence MRI matrices to preoperatively predict tumor biology, chemotherapy response, and, importantly, the likelihood of achieving optimal cytoreductive surgery. By automatically extracting volumetric texture characteristics across the entire peritoneal cavity, these AI-assisted platforms quantify hidden spatial heterogeneity. Advanced radiomics models targeting hidden sites (such as the porta hepatis, lesser omentum, and mesenteric root) can identify subtle non-resectable indicators – such as diffuse superficial encasement of the small bowel or infiltration of the root of the mesentery – that are often missed by visual inspection (45, 46). A dedicated section of literature confirms that deep learning networks fused with clinical indicators significantly outperform standard radiological assessment, providing objective guidance for surgical planning, determining whether primary debulking surgery (PDS) or neoadjuvant chemotherapy (NACT) is preferred, and markedly reducing rates of suboptimal exploratory laparotomy (47, 48).

### 6.3 Assessment of lymph node metastasis

Accurate preoperative assessment of lymph node status is crucial for avoiding unnecessary lymph node dissection and preventing under-staging and inadequate treatment. Currently, the main challenges in CT assessment lie in the missed diagnosis of micrometastatic lymph nodes and the overdiagnosis of inflammatory lymph nodes.A multicenter study by Delvallée et al. showed that CT had diagnostic accuracy rates of 66.7% and 67% for pelvic lymph node and para-aortic lymph node metastases, respectively (49). However, lymph nodes with inflammatory hyperplasia are often misdiagnosed as metastases due to their enlarged size, posing a significant risk of false-positive diagnoses (50). MRI can effectively address these limitations thanks to its functional sequences and superior soft-tissue resolution. The ISAAC prospective study by Fischerova et al. demonstrated that whole-body DWI-MRI and CT are comparable in overall performance for retroperitoneal lymph node staging (with AUCs of 0.76 and 0.72, respectively), and that MRI with DWI sequences can compensate for the limitations of CT, which relies solely on size assessment (51). A systematic review indicates that MRI has higher sensitivity than CT for diagnosing lymph node metastasis (55% vs. 43%), but slightly lower specificity (88% vs. 95%), while PET/CT performs best in terms of both sensitivity and specificity (73% and 97%) (42). However, current techniques still have limitations: Research by Kandemir et al. indicates that imaging methods, including MRI, have an overall sensitivity of less than 45% for ultra-micro lesions smaller than 0.5 cm, and the detection of lymph node metastases is similarly limited (39). Furthermore, the detection rate of lymph nodes in the supra-diaphragmatic region (such as the cardiophrenic angle and the inner mammary region) remains low due to the influence of respiratory movements (51). Future efforts should focus on: establishing standardized scanning protocols and reporting templates (such as the organ-oriented reporting and Node-RADS scoring system recommended by the ESUR guidelines) (50); Develop high-resolution dedicated sequences and breath-holding techniques to improve visualization of specific regions (51); Develop multi-center, large-scale radiomics and deep learning models that integrate lymph node morphology, function (ADC values), and metabolic characteristics to improve the ability to identify ultra-micrometastases (42).

## 7 The application of MRI in evaluating treatment efficacy and predicting prognosis

Given the current clinical challenges of peritoneal metastasis in advanced HGSOC and the development of drug resistance following long-term chemotherapy, the precise assessment of tumor burden and biological behavior is of great significance for developing personalized treatment plans and optimizing patient selection (44). The use of functional MRI effectively addresses the clinical limitations of CT and biochemical markers. First, DWI can sensitively detect peritoneal metastases by quantifying the diffusion of water molecules. A prospective multicenter study has confirmed that the peritoneal cancer index (PCI), based on DWI, is an independent predictor of outcomes following primary tumor cytoreductive surgery (PDS) (52). A study found that an increase in the apparent diffusion coefficient (ADC) of the tumor following one cycle of platinum-based chemotherapy is associated with longer progression-free survival (PFS) in patients (53). The study showed that the mean ADC and certain histogram parameters (such as the skewness coefficient) were significantly higher in the platinum-sensitive group than in the platinum-resistant group, suggesting that high cell density is associated with chemotherapy resistance (54). A prospective study has confirmed that, in patients with elevated CA-125 levels, combined abdominal PET/MRI detects more and smaller peritoneal metastases than PET/CT alone, and has led to a change in treatment strategy for 33% of patients (55).

However, these new technologies still have certain limitations at present. Although there is substantial evidence supporting DWI and PET/MRI, the lack of standardization in scanning protocols, parameters, and analysis methods remains a major obstacle to their widespread adoption across multiple centers (53, 55). Future research should focus on large-scale, multicenter prospective validation studies and promote the standardization of imaging and data analysis. At the same time, further research should explore the clinical applications of new technologies to facilitate their widespread adoption across multiple regions in the future (Table 3).

**TABLE 3:** Summary of representative MRI−based diagnostic models (selected studies).

First author, year	Task	Technique	Main results
Wang et al. (13)	Benign vs. malignant ovarian tumors	CNN combined with clinical variables	Significantly improved the diagnostic performance of junior radiologists to a level comparable to that of senior radiologists
Li et al. (16)	Borderline vs. malignant epithelial tumors	Machine learning	Significantly outperformed radiologists with varying levels of experience
Wei et al. (4)	Type I vs. Type II EOC	Deep learning radiomics nomogram (DLRN)	Significantly superior to single clinical models
Jian et al. (20)	Type I vs. Type II EOC	Four-sequence combined radiomics	Significantly superior to single-sequence models
Cai et al. (21)	Predicting mesenchymal transition (MT) subtype in HGSOC	DWI histogram analysis	Superior to predictions based on single indicators
Lin et al. (24)	MT subtype prediction in HGSOC	T2WI + CE-T1WI radiomics with random forest	The seven selected textural features effectively distinguished the MT subtype from non-MT subtypes
Du et al. (3)	HGSOC vs. CCC	Multi-parametric MRI multi-sequence fusion deep learning	Enabled high-precision preoperative differentiation between HGSOC and OCCC
Shi et al. (31)	PMOC vs. MOMC	Multi-parametric MRI-based radiomics nomogram	Outperformed radiologists
Berardi et al. (40)	MRI-PCI predicting complete debulking	Conventional MRI	High consistency with the mriPCI observer score
Lee et al. (41)	Predicting incomplete debulking	DWI-derived fPCI + ADC + FIGO stage	Accuracy of 92.5%

PCI, peritoneal cancer index; mriPCI, MRI-based peritoneal cancer index. This study lists only a few representative examples; for further data, please refer to the original references.

## 8 Clinical adoption readiness, regulatory barriers, and future roadmap

To facilitate clinical translation and establish an objective framework for future applications, it is essential to categorize current MRI advancements into a clear evidence hierarchy. This structure helps distinguish validated clinical methodologies from experimental pipelines.

### 8.1 Evidence hierarchy and technology readiness levels (TRL)

➀ Universally Validated Clinical Applications (TRL 8-9):

- Conventional Multiparametric MRI (Morphological + DWI/ADC): Highly standardized and globally deployed for differentiating benign from malignant adnexal masses.
- The O-RADS MRI Scoring System: Formally integrated into international guidelines, providing actionable risk stratification and standard reporting templates (such as the organ-oriented reporting and Node-RADS scoring system recommended by the ESUR guidelines).

➁ Advanced Standardizing Modalities (TRL 6-7):

- MRI-based Peritoneal Cancer Index (mriPCI/fPCI): Validated in prospective multicenter clinical trials (e.g., the MISSION trial); highly accurate for predicting complete debulking but requires localized institutional training.

➂ Emerging Research Concepts (TRL 3-5):

- Radiomics Nomograms and Deep Learning Networks: Demonstrate high diagnostic performance under laboratory conditions but are heavily limited by algorithmic opacity and lack of out-of-distribution external validation.
- Molecular Imaging Probes: Dual-responsive nanoprobes and targeted affinity carriers remain in preclinical or small animal testing stages.

### 8.2 Technical implementations and regulatory barriers for AI

The transition of deep learning models from “bench to bedside” is hindered by critical technical and regulatory hurdles.

- **The “Black Box” interpretability problem:** Deep neural networks extract high-dimensional mathematical abstractions that lack direct correlation with established visual pathological criteria (such as cellular atypia, psammoma bodies, or microvessel density). Without reliable visual explanation tools (e.g., advanced Grad-CAM frameworks that correctly localize to the capsule or necrotic core), clinicians are hesitant to trust algorithmic diagnoses for surgical planning.
- **Lack of quantitative data standardization:** Radiomic and deep learning feature extraction is highly sensitive to variations in vendor-specific acquisition parameters, flip angles, and reconstruction filters. Without global harmonization of raw DICOM data, models suffer from severe performance drop-offs when deployed outside their original training centers.
- **Regulatory clearance restrictions:** Currently, very few AI-assisted platforms for ovarian cancer have achieved regulatory clearing certificates [such as FDA 510(k) or NMPA approval]. The absence of clear legal frameworks regarding diagnostic accountability further slows clinical implementation.

### 8.3 Comprehensive future research roadmap

**FIGURE 2:**
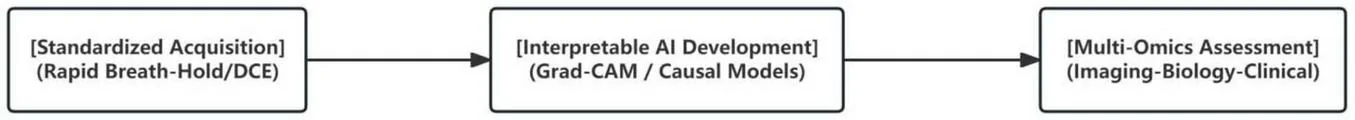
Schematic diagram of the three-stage integrated research roadmap for future ovarian cancer MRI precision diagnosis: standardized rapid breath-hold dynamic contrast-enhanced (DCE) MRI acquisition protocol, interpretable artificial intelligence construction based on Grad-CAM and causal inference models, and multi-omics comprehensive evaluation framework integrating imaging, biological indicators and clinical information. The three modules are executed sequentially to realize the full-process precision diagnosis of ovarian cancer.

To guide future research priorities and maximize the clinical impact of advanced imaging, we propose a tripartite technical roadmap: (Figure 2)

**➀ Technical optimization:** Develop rapid breath-hold sequences and bowel artifact suppression sequences to improve the detection rate of microlesions (<0.5 cm) in hidden peritoneal niches like the small bowel mesentery and the diaphragmatic dome.

**➁ Algorithmic transparency:** Shift AI research away from uninterpretable, single-center retrospective architectures toward multicenter, prospective designs utilizing physics-informed neural networks and explicit causal models.

**➂ Multi-omics synthesis:** Construct a fully integrated “imaging-pathology-clinical” diagnostic framework. Future paradigms must combine quantitative MRI radiomics with longitudinal serum biomarker tracks and high-throughput liquid biopsy genomics to achieve absolute non-invasive precision medicine.

## 9 Summary and outlook

In summary, MRI has evolved from traditional morphological descriptions to a comprehensive multimodal assessment system that integrates multi-parametric functional imaging, radiomics, and deep learning technologies in the precision diagnosis and treatment of ovarian cancer. Existing research has made significant progress in distinguishing between benign and malignant tumors, classifying pathological subtypes, staging the disease, evaluating treatment efficacy, and predicting prognosis, thereby establishing the critical role of multi-parameter MRI and artificial intelligence models in diagnosing this disease and developing personalized treatment plans.

However, current research – particularly in emerging fields such as AI – still faces common challenges, including limited model generalizability, poor interpretability, and low detection rates for micro-lesions. Future research should focus on standardizing MRI acquisition and data protocols, encourage large-scale, multicenter prospective studies, and explore the potential for integrating interpretable AI with molecular imaging probes. The goal is to establish an integrated “imaging-pathology-clinical” precision assessment system, thereby achieving a transition from merely “detecting lesions” to “analyzing lesions,” and comprehensively improving the level of preoperative precision diagnosis and treatment for ovarian cancer.
